# Body mass index and depressive symptoms in middle aged and older adults

**DOI:** 10.1186/s12889-015-1663-z

**Published:** 2015-03-31

**Authors:** Jin-Won Noh, Young Dae Kwon, Jumin Park, Jinseok Kim

**Affiliations:** Department of Healthcare Management, Eulji University, Seongnam, Korea; Department of Humanities and Social Medicine, College of Medicine and Catholic Institute for Healthcare Management, the Catholic University of Korea, Seoul, Korea; University of Maryland School of Nursing, Baltimore, MD USA; Department of Social Welfare, Seoul Women’s University, Rm. #721, Insa-Kwan Bldg., 126 Kongneung 2-Dong, Nowon-Ku, Seoul, Korea

**Keywords:** Body mass index, Depressive symptoms, U-shaped relationship, Middle aged and older adults, Korean Longitudinal Study of Aging (KLoSA)

## Abstract

**Background:**

The relationship between weight problems and depression has been the focus of many studies; however, results from these studies vary. The purpose of this study is to describe the association between depression and BMI using data from a national sample of middle aged and older Koreans and to examine whether gender moderates the relationship between depression and weight.

**Methods:**

We used data from the Korean Longitudinal Study of Aging (KLoSA). Of the 7,920 respondents that participated in KLoSA in 2010, 7,672 adults aged between 50 and 102 years were included in the final analysis. The relationship between depression and obesity status was examined in both the full sample and in sub-samples stratified by gender. The observed U-shaped association between obesity status and CES-D score was tested by regressing CES-D score on linear and quadratic terms of BMI scores.

**Results:**

The distribution of CES-D scores by respondents’ obesity status (i.e., underweight, normal weight, overweight, obese and severely obese) showed a U-shaped association. Specifically, the highest CES-D scores were found in underweight individuals; this was followed by the severely obese and obese groups in the full sample and in gender-specific subsamples. The lowest CES-D scores were found in the overweight group when considering the entire population and males alone and in the normal weight group for females. This U-shaped association between CES-D and obesity status was confirmed by a model in which CES-D scores were regressed on BMI scores and other covariates.

**Conclusions:**

This study found a U-shaped association between BMI and levels of depressive symptoms among adults in Korea overall and also within each gender. Specifically, the highest level of depressive symptoms was found among the underweight, followed by the severely obese and then the obese. Slightly different patterns between male and female adults were found regarding the weight status associated with the fewest depressive symptoms.

## Background

Both depression and weight problems are common, and they are increasing public health concerns [1-3]. Depression is estimated to affect 350 million people, according to the World Health Organization [4]. Since 1980, the prevalence of obesity has been increasing steadily worldwide and has nearly doubled [5]. Therefore, the burden of disease and the associated economic costs stemming from depression and obesity are great [4,5].

In recent years, the relationship between weight problems and depression has been the focus of much research; however, the results from current studies are inconsistent. Both positive [6-9] and negative [10,11] associations have been found; in addition, several studies found no association [12,13]. These results suggest that not all people in the general population whose weight is outside the recommended range experience psychological problems such as depression, and there could be specific factors affecting certain individuals. Socio-demographic factors are potential moderators and mediators of the relationship between depression and weight problems and may explain the controversial findings [14].

Gender has been hypothesized as a potential moderator of the relationship between depression and obesity. However, there are conflicting results with respect to this as well. Several studies found that obesity was a significant predictor of depression in females but not in males [15-17]. In contrast, a large population-based study in adults found a significant depression-obesity relationship in males but not in females [18]. McCrea et al. [6] reported that there was a positive association between mental disorders and Body Mass Index (BMI) in young women, whereas the relationship in young men was U-shaped; the risk of depression was higher in both obese and underweight males. Other studies found no gender discrepancy [8,12].

The relationship between weight and depression may be different in middle-aged and older adults compared to younger adults because functional limitations and medical comorbidities related to aging may lead to weight change and also be associated with mood change [12]. However, many cross-sectional studies exploring the association between BMI and depression excluded elderly people. Furthermore, several studies that focused on people aged 50 years and over suggested contradictory results [8,12,17]. In addition, the majority of them sampled Western populations, and few studies were conducted in Asian populations [13,19]. Therefore, a population-based study of the Korean elderly may be helpful in clarifying the association between BMI and depression. In addition, most studies were focused on overweight individuals; therefore, there is a need for a survey on both overweight and underweight individuals to be conducted. This analysis explores the full range of BMI classes, overweight or obese, normal weight, and underweight, and is stratified by gender. The purpose of this study is to evaluate the association between depression and BMI in a large representative sample of middle aged and older Korean individuals and examine whether the relationship between depression and weight differs by gender.

## Methods

### Data and subjects

We used data from the Korean Longitudinal Study of Aging (KLoSA), which was obtained from a public repository [20]. The Korean Labor Institute conducted KLoSA, which was funded by the Korean Ministry of Labor. Data were collected every other year beginning in 2006. There were 7,920 respondents who participated in Wave 3 of the KLoSA in 2010; descriptive statistics regarding the respondents are presented in Table 1. Among these, there were 245 respondents who either did not answer questions regarding depression (n = 52) or did not have available BMI data (n = 195). Additionally, educational data were not available for three respondents, which resulted in 7,672 cases that were included in the final regression analysis. This study was approved by the Institutional Review Board of the Catholic University of Korea; we received a waiver of informed consent because the data were obtained from a public database.

**Table 1:** **Characteristics of study sample stratified by gender**

**Variable**	**Total**		**Male**		**Female**	
	**(N = 7,920)**		**(N = 3,412)**		**(N = 4,508)**	
	**Mean**	**(SD)**	**Mean**	**(SD)**	**Mean**	**(SD)**
Age (years)	66.3	(10.6)	65.8	(10.0)	66.6	(10.9)
CESD scores	7.53	(5.64)	8.02	(5.80)	6.89	(5.35)
	N	%	N	%	N	%
Marital status						
Married	6,143	77.6	3,120	91.4	3,023	67.1
Not married	1,777	22.4	292	8.6	1,485	32.9
Education						
Elementary school or lower	3,723	47.0	1,074	31.5	2,649	58.8
Middle school	1,328	16.8	600	17.6	728	16.2
High school	2,110	26.7	1,174	34.4	936	20.8
College or higher	756	9.6	563	16.5	193	4.3
Employed						
Not employed	4,523	57.1	1,417	41.5	3,106	68.9
Employed	3,397	42.9	1,995	58.5	1,402	31.1
Self-rated health status						
Very bad	448	5.7	167	4.9	281	6.2
Bad	1,937	24.5	648	19.0	1,289	28.6
Neutral	2,949	37.2	1,209	35.4	1,740	38.6
Good	2,407	30.4	1,290	37.8	1,117	24.8
Very good	179	2.3	98	2.9	81	1.8
Obesity status						
Underweight	349	4.5	151	4.5	198	4.5
Normal	3,382	43.8	1,439	42.9	1,943	44.4
Overweight	2,225	28.8	1,094	32.6	1,131	25.9
Obese	1,663	21.5	641	19.1	1,022	23.4
Severely obese	106	1.4	28	0.8	78	1.8

### Variables

#### Dependent variables

The presence of depressive symptoms was assessed by the Korean version of the Center for Epidemiologic Studies Depression Scale (CES-D) survey. The CES-D 10, a simplified form of the CES-D, is a questionnaire composed of 10 questions and was developed as a depressive symptom screening scale for epidemiological investigations [21]. In the Korean version of the CES-D 10, answers to questions regarding the frequency of experiencing depressive symptoms during the past week were composed of four choices (0 = ‘occasionally’ (less than one day); 1 = ‘sometimes’ (from one to two days); 2 = ‘often’ (from three to four days); 3 = ‘at all times’ (from five to seven days). A depression score was obtained by calculating the total score of the 10 items, which ranged from 0 to 30; higher scores indicated more severe depressive symptoms (Cronbach’s alpha = .861 in this sample).

#### Independent variables

BMI is commonly used to assess body fat composition and was defined as weight in kilograms divided by height in meters squared (kg/m^2^). BMI values were calculated with self-reported weight and height. In this study, participants were classified as underweight, normal weight, overweight, obese or severely obese based on World Health Organization Western Pacific Region suggested revised Asia-Pacific criteria (less than 18.5 kg/m^2^, between 18.5 kg/m^2^ and 23 kg/m^2^, between 23 kg/m^2^ and 25 kg/m^2^, between 25 kg/m^2^ and 30 kg/m^2^ and more than 30 kg/m^2^, respectively).

Other covariates were defined using a questionnaire from the KLoSA. Marital status was classified into two groups: married and not married (i.e., divorced, parted by death, separated or single). Level of education was divided into four groups: college or higher, high school graduate, middle school graduate and elementary school or lower. Employment status was either employed or unemployed. Participants were asked to rate their health status on a five-point Likert scale (1 corresponding to “very good” and 5 to “very bad”).

### Statistical analysis

Descriptive analyses were conducted to provide summary statistics of the sample characteristics. Due to gender differences in the relationship between depression and obesity status, results of a gender-stratified analysis were also provided. The relationship between depression and obesity status for the overall sample and the single gender samples were examined using a set of graphs. We have tested the significance of the interaction between gender and obesity status on CES-D score. The U-shaped association between obesity status and CES-D score was tested using a regression model of CES-D score on linear and quadratic terms of BMI scores.

## Results

Socio-demographic characteristics of the study participants are presented in Table 1. There were 7,920 adults aged between 50 and 102 years (Mean (SD) = 66.3 (10.6)) included in this study; 4,508 (56.9%) were female. Over three-quarters (77.6%) of the participants were married. Almost half the participants (47%) reported having no education past elementary school; 17% were middle school graduates, 27% were high school graduates, and only one out of 10 had a college or graduate degree. Fewer than half (43%) were employed at the time of study. Less than a third of the participants reported their health status as very bad (6%) or bad (25%), while 30% reported good and 2% very good health. Socio-economic characteristics such as age (t = 3.23, p = .001), marital status (*χ*^*2*^(1) = 663.50, p < .001), education level (*χ*^*2*^(3) = 749.45, p < .001), employment status (*χ*^*2*^(1) = 593.94, p < .001), self-rated health status (*χ*^*2*^(4) = 203.01, p < .001), and obesity status (*χ*^*2*^(4) = 59.55, p < .001) were significantly different by gender.

**Figure 1:**
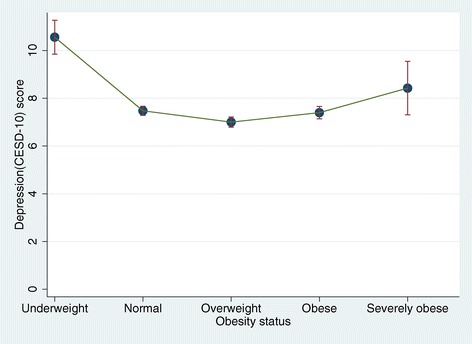
**Distribution of CESD-10 scores by obesity status.**

**Figure 2:**
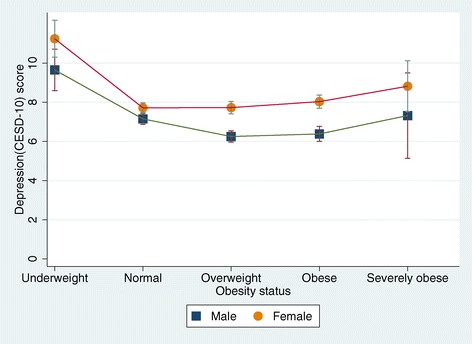
**Distribution of CESD-10 scores by obesity status and gender.**

Level of depressive symptoms measured by CES-D 10 was higher for female (Mean (SD) = 8.02 (5.80)) than for male (Mean (SD) = 6.89 (5.35)) (Table 1). Figure 1 shows the overall relationship between depression and obesity status. The CES-D 10 measure of depression was highest among those who were underweight (Mean (SD) = 10.6(6.7)) followed, in order, by the severely obese (Mean (SD) = 8.4(5.9)), normal (Mean (SD) = 7.5(5.6)) and obese (Mean (SD) = 7.4(5.4)) groups; depression was lowest among overweight individuals (Mean (SD) = 7.0(5.2)). The differences between the CES-D 10 scores of the different BMI groups were significant (F(4, 7670) = 32.2, p < .001); this held for both males (F(4, 3336) = 17.0, p < .001) and females (F(4, 4329) = 18.4, p < .001). As shown in Figure 2, however, the distribution of CES-D 10 scores by obesity status differed significantly between genders (F(4, 7665) = 3.8, p = .004). Specifically, the least depressed group of males consisted of those who were overweight (Mean (SD) = 6.3(4.9)); in contrast, for females, the least depressed group consisted of those in the normal weight range (Mean (SD) = 7.7(5.4)).

Figures 1 and 2 also show that the association between obesity status (i.e., underweight, normal weight, overweight, obese and severely obese) and depression was U-shaped. Specifically, the highest CES-D scores were found in the underweight and severely obese groups while the lowest were in the overweight group for the total and male samples and in the normal weight group for females.

Results from a model of CES-D score regressed on linear and quadratic BMI scores are summarized in Table 2. After controlling for socio-demographic and health-related characteristics including gender, age, marital status, education level, employment status, and self-rated health status, the quadratic effect of BMI on CES-D score was positive and significant [B(S.E) = 0.020(0.005), p < .001]; this was found in models for both males [B(S.E) = 0.015(0.008), p = .045] and females [B(S.E) = 0.022(0.006), p < .001] (Table 2). These results were consistent with the findings presented in Figure 1.

**Table 2:** **Regression analysis of CES-D 10 on BMI, BMI-squared, and other covariates stratified by gender**

	**Total (N = 7,672)**				**Male (N = 3,340)**				**Female (N = 4,332)**			
	**B**		**SE (B)**	**95% CI**	**B**		**SE (B)**	**95% CI**	**B**		**SE (B)**	**95% CI**
Gender												
Male (reference)												
Female	−0.19		0.13	(−0.44, 0.07)								
BMI	−1.01	^***^	0.22	(−1.44, −0.58)	−0.81	^*^	0.35	(−1.5, −0.12)	−1.10	^***^	0.28	(−1.65, −0.55)
BMI-squared	0.02	^***^	0.00	(0.01, 0.03)	0.02	^*^	0.01	(0, 0.03)	0.02	^***^	0.01	(0.01, 0.03)
Age	0.01		0.01	(−0.01, 0.02)	−0.01		0.01	(−0.03, 0.01)	0.02		0.01	(0, 0.04)
Education												
Elementary school or lower (reference)												
Middle school graduate	−0.52	^**^	0.17	(−0.86, −0.18)	−0.77	^**^	0.25	(−1.26, −0.28)	−0.28		0.24	(−0.74, 0.18)
High school graduate	−0.73	^***^	0.16	(−1.04, −0.41)	−0.64	^**^	0.22	(−1.07, −0.21)	−0.80	^**^	0.23	(−1.26, −0.34)
College or higher	−1.37	^***^	0.22	(−1.81, −0.94)	−1.65	^***^	0.26	(−2.17, −1.14)	−0.79		0.41	(−1.59, 0.01)
Employment status												
Not employed (reference)												
Employed	−0.86	^***^	0.13	(−1.12, −0.6)	−1.68	^***^	0.29	(−2.26, −1.11)	−0.73	^***^	0.19	(−1.11, −0.36)
Marital status												
Not married (reference)												
Married	−1.04	^***^	0.15	(−1.34, −0.74)	−1.31	^***^	0.20	(−1.69, −0.92)	−0.54	^**^	0.18	(−0.9, −0.18)
Self-rated health status	−2.07	^***^	0.07	(−2.21, −1.93)	−1.89	^***^	0.10	(−2.08, −1.69)	−2.20	^***^	0.10	(−2.39, −2.01)

Note: *p < .05, **p < .01, ***p < .001; B: regression coefficient; The Center for Epidemiologic Studies Depression Scale (CES-D), Body Mass Index (BMI).

## Discussion

In this study, we analyze the relationship between obesity, measured by BMI, and depression, measured by the CSE-D 10. As a result, we revealed that there was a relationship between BMI and depressive symptoms; however, it was not linear but U-shaped. In addition, through multiple regression analyses which controlled for key socio-demographic variables, we confirmed that BMI had a quadratic curve linear relationship with depression.

Until now, most results from studies that analyzed the relationship between obesity and depression revealed a positive one [7,9,22,23]. This means higher levels of depression are associated with increased obesity. However, there were several studies that demonstrate a negative a relationship (i.e., higher levels of depression were associated with lower levels of obesity), no association or mixed results [11-13]. While most studies from western countries showed that there is a positive relationship between obesity and depression, in Asian countries such as Japan, Taiwan and Hong Kong their studies showed negative relationship from the elderly [24-26]. A possible explanation for the varying results is that variables such as gender, age, socioeconomic status, ethnic group, nationality, health status, etc. affect the relationship between obesity and depression [27].This study identified a key finding that obesity status was associated with depressive symptoms but the relationship was not linear. Severely obese and underweight individuals exhibit high degrees of depressive symptoms; this is especially true in the underweight group. The overweight group rests at the bottom of the U-shaped relationship. There are previous studies that have demonstrated the relationship between BMI and depression is U-shaped [6,23,28,29]. Despite the fact that the nationality, age range and ethnic group represented in this sample were different from those in previous studies, this study confirmed the same result. Furthermore, unlike previous studies that showed only non-linear relationship using a graph, we tested and found that the U-shaped relationship between BMI and depressive symptoms was valid even after controlling for socio-economic variables. In a future research, other mental illnesses should be explored because previous studies have demonstrated that not only depression but also other mental illnesses are associated with obesity [6,30].

In this study, we found overweight group had the lowest score of depressive symptoms. It implies we might have to reconsider the current understanding about the relationship between overweight and health status, which views being overweight as not only a precursor to obesity but also as a critical risk factor of poor health outcomes. Several recently released studies found that health status in the overweight group was the same as or better than in the normal weight group. They suggest that standards for obesity and management of the overweight group may need to be changed [31-34].We draw attention to the fact that depressive symptom were highest in the underweight group. With the rise of obesity in the population, social interest and healthcare policies are focused on the obesity problem; however, we should also pay attention to the health problems of the underweight group. The portion of the population that is underweight, particularly among seniors and adolescents, is not small and their health problems are severe in developed countries [35-38]. Because our analysis did not control for all potential confounders of the relationship between underweight and depressive symptoms, future studies are warranted that include further controls for socioeconomic status and health.

In this study, the relationship between BMI and depressive symptoms differed by gender. Previous studies that analyzed the relationship between obesity and depression revealed that it differed by gender [17,20,39]. This may be explained by the fact that physical, socioeconomic and environmental factors affect men and women differently [40]. In this study, the reason that women in the normal weight group had the lowest degree of depressive symptoms, lower than that of the overweight group, may be related to the fact that social interest in women’s weight and appearance is exceptionally high in Korean society. Therefore, women are under strong pressures and exhibit stress related to weight [41].

This study is limited by its cross-sectional nature and its target age group, seniors over 50 years of age; therefore, future studies that analyze the relationship between obesity and depression in a wider range of subjects are warranted. In particular, certain answers should be obtained via a longitudinal approach that analyzes the influence of weight change on mental health. The fact that obesity is only measured by BMI is another limitation of this study. BMI data could be under or over reported because they were self-reported. Considering the limitations of BMI, future studies that analyze the relationship between obesity and depression via other metrics are needed; other indicators of obesity include waist measurement and thigh circumference.

## Conclusions

In this study, we analyzed the relationship between BMI and depressive symptoms, as measured by the CSE-D 10, in a target population of middle-aged and older people (i.e., those >50 years of age). We controlled for key socio-demographic variables and confirmed that BMI had a quadratic effect on depressive symptoms. The depressive symptoms in the underweight and severely obese groups were highest. In contrast, the normal weight and overweight groups had the lowest depression levels, and there was a difference between men and women. This study shows that we need to focus more on the mental health of low-weight middle aged and older people beyond former viewpoint of positive association between obesity and depression. Future studies regarding the association between changes in weight and depressive symptoms are needed for causality.
